# Diffuse astrocytoma, AYA-type, frequently MAPK-altered: report of 45 patients

**DOI:** 10.1007/s00401-025-02873-8

**Published:** 2025-04-09

**Authors:** Omkar Singh, Christopher Dampier, Zied Abdullaev, Karen Dazelle, Hye-Jung Chung, Kyle Conway, Sandra Camelo-Piragua, Stewart Neill, Daniel Brown, James Stephen Nix, Caterina Giannini, Robert Macaulay, Daniel Marker, John Skaugen, Scott Kulich, Han Lee, Orwa Aboud, Peter Pytel, Ewa Borys, Arie Perry, Laila Naqib-Osman, Igor Lima Fernandes, Qinwen Mao, Mouied Alashari, Cheddhi Thomas, Jeffrey Helgager, Maria A. Gubbiotti, John Newman, Nishant Tiwari, Patrick J. Cimino, Martha Quezado, Kenneth D. Aldape, MacLean P. Nasrallah

**Affiliations:** 1 Laboratory of Pathology, Center for Cancer Research, National Cancer Institute, National Institutes of Health, Bethesda, MD USA; 2 Department of Pathology, University of Michigan, Ann Arbor, MI USA; 3 Department of Pathology and Laboratory Medicine, Emory University School of Medicine, Atlanta, GA USA; 4 CellNetix Laboratories, Seattle, WA USA; 5 Department of Medical Education, Alice L. Walton School of Medicine, Bentonville, AR USA; 6 Department of Laboratory Medicine and Pathology, Mayo Clinic, Rochester, MN USA; 7 Department of Anatomic Pathology, Moffitt Cancer Center, Tampa, FL USA; 8 Department of Pathology, University of Pittsburgh School of Medicine, Pittsburgh, PA USA; 9 Department of Pathology and Laboratory Medicine, Comprehensive Cancer Center, University of California- Davis, Sacramento, CA USA; 10 Department of Pathology, University of Chicago, Chicago, IL USA; 11 Department of Pathology and Laboratory Medicine, Loyola University, Maywood, IL USA; 12 Departments of Pathology and Neurological Surgery, University of California, San Francisco, San Francisco, CA USA; 13 Lower Columbia Pathologists, Longview, WA USA; 14 Laboratorio Bacchi, Botucatu, Brazil; 15 Department of Pathology, University of Utah School of Medicine, Salt Lake City, UT USA; 16 Department of Pathology, Inova Fairfax Hospital, Fairfax, VA USA; 17 Department of Pathology, School of Medicine and Public Health, University of Wisconsin, Madison, WI USA; 18 Department of Pathology, University of Texas MD Anderson Cancer Center, Houston, TX USA; 19 Department of Pathology, Stanford University, Stanford, CA USA; 20 Department of Pathology, Phoenix Children’s Hospital, Phoenix, AZ USA; 21 Surgical Neurology Branch, Disorders and Stroke, National Institute of Neurological, National Institutes of Health, Bethesda, MD USA; 22 Department of Pathology and Laboratory Medicine, Hospital of the University of Pennsylvania, Perelman School of Medicine, Philadelphia, PA USA; 23 Department of Biomedical and Neuromotor Sciences (DIBINEM), Alma Mater Studiorum, University of Bologna, Bologna, Italy

**Keywords:** Methylation profiling, Epigenetics, Brain tumors, Glioma, MAPK, AYA, HGG_B

## Abstract

A putative molecular subtype of IDH-wildtype diffuse glioma with recurrent MAPK pathway alterations has recently been reported. By dimensionality reduction analysis of genome-wide methylation profiling, these tumors form a distinct methylation cluster of gliomas. Characterization of 47 tumors from 45 patients reveals that these gliomas are predominantly supratentorial in young adults, are highly infiltrative, and harbor mitogen-activated protein kinase (MAPK) pathway alterations with high rates of *CDKN2A/2B* deletion, *PDGFRA* amplification, *MYCN* amplification, *NF1* variants, and *BRAF* alterations. The tumors’ epigenetics are distinct from other adult and pediatric gliomas in the 2021 World Health Organization (WHO) classification. The histology of the gliomas most often demonstrates high-grade astrocytic features, but can be variable from tumor to tumor, as well as fall into a spectrum of histologic grades. Outcomes show considerable variability based on histologic grade and molecular features, supporting grading within this group of tumors to ensure optimal care choices on an individual patient basis. These unifying epigenetic, sequencing, and infiltrative astrocytic features allow the tumors to be considered diffuse astrocytoma, adolescent, and young adult-type, with MAPK alterations (DAYA).

**Supplementary Information:**

The online version contains supplementary material available at 10.1007/s00401-025-02873-8.

## Introduction

DNA methylation is an epigenetic marker with a role in modification of chromatin structure, gene expression, and genome stability [3, 11]. Recent advances in central nervous system (CNS) tumor classification based on DNA methylation profiling provide the opportunity to characterize epigenetic subtypes of existing tumors [4, 13, 17] and to explore newly observed epigenetic clusters of tumors [12, 14, 20]. Investigation of DNA methylation-based tumor classes has prompted the integration of newly identified tumor types and subtypes into the 2021 edition of the World Health Organization (WHO) classification of central nervous system (CNS) tumors [22]. Here, using methodologies previously described [1, 5, 24] and following a recently published manuscript reporting 32 cases of IDH-wildtype adult-type diffuse glioma with recurrent MAPK pathway alterations and a distinct methylation profile [19], we characterize 47 additional tumors from 45 patients (2 patients with primary-recurrent pairs) that matched to the same methylation class, designated “adult-type diffuse high-grade glioma, IDH-wildtype, subtype B” (HGG_B)” on the v12 Heidelberg methylation classifier. Tumors in this methylation cluster represent a diffuse adolescent and young adult-type (AYA-type) astrocytoma and are frequently MAPK-altered (“diffuse astrocytoma, AYA-type, MAPK-altered,” DAYA).

## Materials and methods

### Data collection

The use of human subject material was performed in accordance with the World Medical Association Declaration of Helsinki and with the approval of the participating Institutional Review Boards. A cohort of 45 patients (47 samples, 2 pairs of primary and recurrent, Supplementary Table 1) classified as adult-type diffuse high-grade glioma B subtype (HGG_B) with calibrated scores greater than 0.9 using the DKFZ classifier, version v12b6 [4]. A majority of the samples (*n* = 30) were processed at the NIH; 7 samples were processed at other locations and idat files were submitted to the NCI clinical methylation unit for classification. In addition, 10 samples were collected from publicly available data [4, 7, 10, 18, 21, 23]. Sequencing and clinical data were collected from the respective data sources. A heatmap derived from SNP probes verified uniqueness of the samples, with the only two sets of duplicates representing two patients for whom both primary and recurrent tumors were studied (Supplementary Fig. 1).

### Methylation profiling

All NCI samples (*n* = 30) were profiled using bisulfite-converted genomic DNA applied to Infinium MethylationEPIC and MethylationEPICv2 kits (Illumina, USA) as part of clinical methylation testing and analyzed by the DKFZ classifier v12b6 as described by Capper et al. [4]. A subset of the external and publicly available samples was profiled using the 450 Illumina array; therefore, overlapping probes (*N* = 357,483) among all three types of arrays were utilized to process samples uniformly. All SNP linked probes were removed before analysis. The “minfi” R package was utilized to process all samples and to estimate beta values for each probe [2]. To create copy-number variation (CNV) profiles, the R “conumee” package with default parameters along with normal brain samples (http://bioconductor.org/packages/conumee/) was employed.

### UMAP and unsupervised hierarchal clustering

The uniform manifold approximation and projection (UMAP) was created with the inclusion of in-house cohorts of GBM_MES_TYP (*n* = 103), GBM_RTK_I (*n* = 93), GBM_RTK_II (*n* = 94), HGAP (*n* = 83), DAYA (*n* = 47), pedHGG_A (*n* = 10), pedHGG_B (*n* = 8), pedHGG_MYCN (*n* = 26), pedHGG_RTK1A (*n* = 52), pedHGG_RTK1B (*n* = 24), pedHGG_RTK1C (*n *= 34), pedHGG_RTK2A (*n* = 25), pedHGG_RTK2B (*n* = 20), ANTCON/GTAKA (6), GG (94), HPAP (27), PA_CORT (81), PA_MID (100), PA_PF (108), PLNTY (8), and PXA (121). Full names are provided in Supplementary Table 2. The raw idat files were processed using the “minfi” package with the single sample Noob (ssNoob) normalization method to obtain beta values. Prior to clustering, probes (*n* = 152,826) with SNP extension, probes from X and Y chromosomes, and probes where the 5 bp 3′-subsequence (including extension for type II) overlapped with any of the SNPs with global MAF > 1% were removed [25]. Only 450 k/EPIC/EPICv2 overlapped probes (*n* = 357,483) were selected; the 20 principal components (PCs) using the most variable 20 k probes across all classes were calculated and subsequently employed to create the UMAP. Unsupervised clustering was performed on the PC matrix using the umap function of the uwot R package with the following non-default parameters: n_neighbors = 10, n_components = 4, metric = "cosine", min_dist = 0, spread = 1. Similarly, unsupervised hierarchical clustering was performed on the relevant classes using the most variable 5 k probes. The *ComplexHeatmap* R package was utilized to generate heatmaps and perform hierarchical clustering [6].

### Differential methylation promoter (DMP) analysis

To assess the methylation status of promoters, M values were calculated for each probe, and then, all probes present in the transcriptional start site TSS1500 and TSS200 regions were selected. The median M value for all probes of a gene was estimated to represent promoter methylation status. After calculating promoter methylation status, the methylation status of promoters in DAYA samples was compared to those of glioblastomas and pediatric high-grade gliomas using the “limma” R package for DMP analysis [9]. Differential change in promoters was considered when the *p* value was < 0.05. A threshold of -Log10p (20) and onefold change were set as representing a significant change in promoter methylation status; the “EnhancedVolcano” R package was used to create volcano plots (https://github.com/kevinblighe/EnhancedVolcano).

### Survival analysis

The survival data were collected from the original source of data when available, as described in data collection. Kaplan–Meier survival analyses were performed with the log-rank test using the survminer and survival packages in R (https://github.com/kassambara/survminer, https://CRAN.R-project.org/view=Survival). Diagnoses for TCGA cohorts were verified by utilizing only cases for which DNA methylation scores for their respective diagnoses were greater than 0.9.

### Copy-number variation analysis

For copy-number variation (CNV) analysis, we processed all array data using the minfi R package and performed CNV analysis using the conumee R package (https://bioconductor.org/packages/release/bioc/html/conumee.html). According to the conumee documentation, the overall intensity of each query sample is compared against a set of control samples, and the log2 ratio of probe intensities within predefined genomic bins was calculated. To establish the copy-number neutral state, intensity values were adjusted to minimize the median absolute deviation of all bins to zero.

The analysis included samples from three types of Illumina arrays: 450 k, EPIC, and EPICv2, CBM_RTK_II (*n* = 94), HGAP (*n* = 83), pedHGG_MYCN (26), pedHGG_RTK1A (*n* = 52), pedHGG_RTK1B (*n* = 24), pedHGG_RTK1C (*n* = 34), pedHGG_RTK2A (*n* = 25), and pedHGG_RTK12B (*n* = 20). To streamline CNV analysis across these platforms, we utilized overlapping probes across all arrays. For the summary copy-number plots, segment ratios were calculated for each sample, followed by visualization using the GenVisR package (https://bioconductor.org/packages/release/bioc/html/GenVisR.html). A CNV cutoff of ± 0.1 was applied using the CN_Freq() function to define low and high copy-number changes.

## Results

### Identification of an epigenetically distinct infiltrating astrocytoma of young adults harboring MAPK pathway alterations

**Fig. 1:**
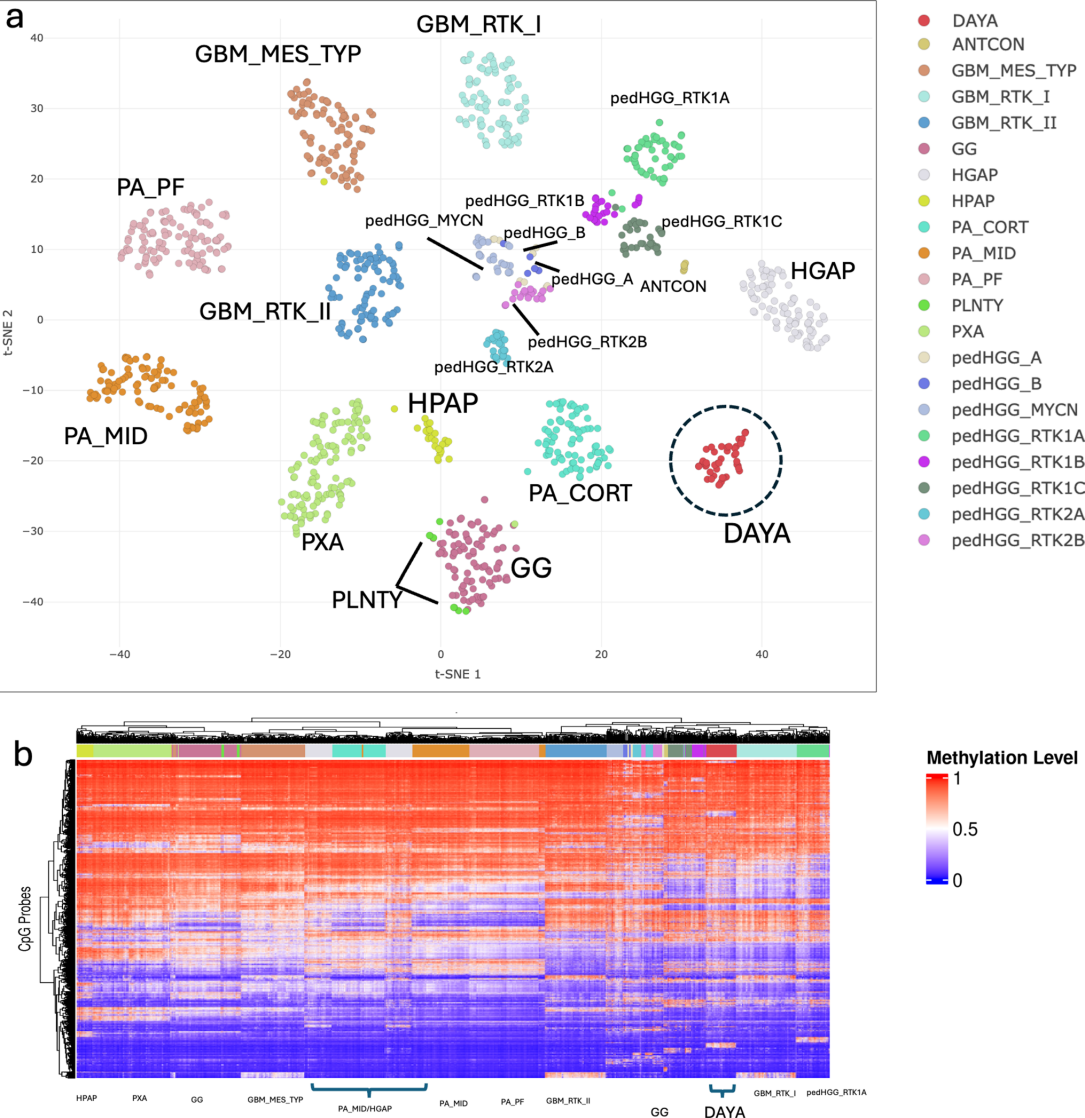
The methylation cluster DAYA is epigenetically distinct from tumor types in the 2021 WHO classification. **a** Unsupervised clustering of DNA methylation data using UMAP embedding (*n* = 10,000, most variable probes). Samples from this proposed group (DAYA, *n* = 48) were embedded with relevant selected CNS tumors from the in-house cohort: GBM_MES_TYP (*n* = 103), GBM_RTK_I (*n* = 93), GBM_RTK_II (*n* = 94), HGAP (*n* = 83), DAYA (*n* = 47), pedHGG_A (*n* = 10), pedHGG_B (*n* = 8), pedHGG_MYCN (*n* = 26), pedHGG_RTK1A (*n* = 52), pedHGG_RTK1B (*n* = 24), pedHGG_RTK1C (*n* = 34), pedHGG_RTK2A (*n* = 25), pedHGG_RTK2B (*n* = 20), ANTCON/GTAKA (6), GG (94), HPAP (27), PA_CORT (81), PA_MID (100), PA_PF (108), PLNTY (8), and PXA (121). All samples were classified by the DKFZ classifier with scores > 0.9. **b** Unsupervised hierarchical clustering of DNA methylation data from the same set of tumors with differentially methylated 5000 probes

A group of IDH-wildtype gliomas that did not correspond to an established tumor type in the 2021 WHO classification consistently demonstrated tight clustering on dimensionality reduction analysis and matched to the “Adult-type diffuse high-grade glioma, IDH-wildtype, subtype B” class in the Heidelberg version 12.8 classifier. Based on both clinical and genomic features, we have proposed the name “Diffuse astrocytoma, AYA-type, frequently MAPK-altered (DAYA) for this methylation class. The cohort analyzed here consisted of 47 tumors in this methylation cluster, 37 of which are newly profiled, with the remaining 10 available from online sources. A UMAP using unsupervised clustering depicts the tight grouping of this cohort (*n* = 47) and the relationship of the cluster to other CNS tumor types (*n* = 586), separate on this analysis from other glioma methylation classes (Fig. 1a). In addition, unsupervised hierarchical clustering based on beta values from the 5000 most variable probes used in the DNA methylation profiling illustrates the relationships of the DAYA cluster to other gliomas (Fig. 1b). The DAYA tumors clustered tightly together and separate from other gliomas and showed a methylation profile on the heatmap that is remarkably consistent among all DAYA cases, and contrasts with the profiles of other tumor types. Specifically, compared to glioblastomas (GBMs), many CpG sites that are hypermethylated in GBMs are hypomethylated in DAYA, and vice versa. Relative to high-grade astrocytomas with piloid features (HGAPs), many methylation probes show similarities, but overall the two methylation clusters show differences in their patterns. The profile of DAYA appears more similar to that seen in pediatric high-grade gliomas (pedHGG), albeit distinct even from this family of tumors, and clustering separately. Analysis of gene promoters that are differentially methylated among tumor types verified the distinct nature of the epigenetic features of the DAYA methylation cluster in comparison to HGAP, pedHGG, and GBM (Supplementary Fig. 2). DAYA had subsets of both hypermethylated and hypomethylated promoters relative to both HGAP and GBM; compared to pedHGG, promoters that were hypermethylated were more prominent in DAYA. The *MGMT* promoter was not methylated in 46/48 cases, a rate of methylation not seen in GBM or HGAP, but closer to the rate in pedHGG [8].

**Fig. 2:**
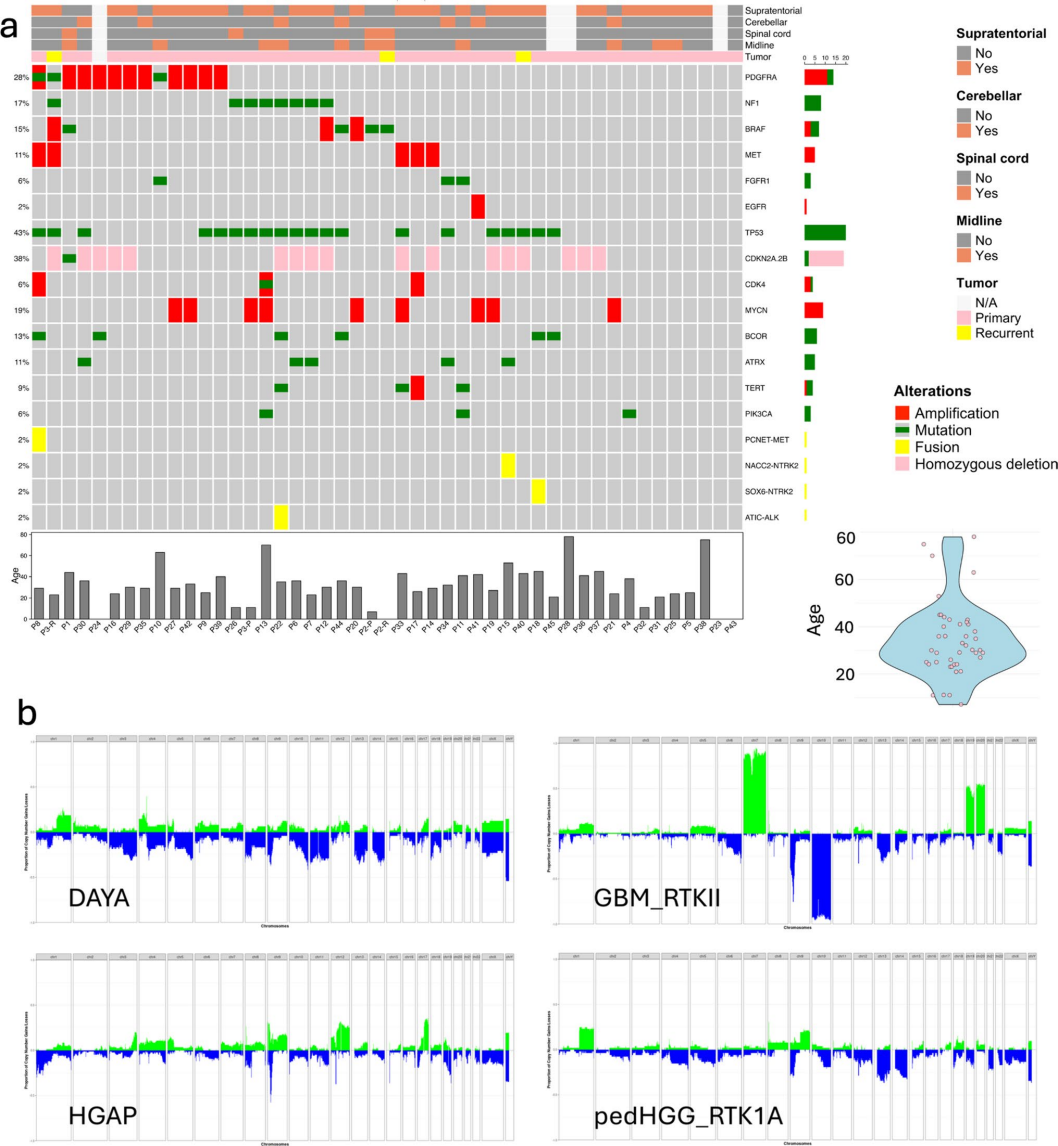
**a **Oncoplot of DAYA. Tumor locations are indicated at the top, followed by whether each tumor is a primary or recurrent glioma. The genomic findings highlight involvement of the MAPK pathway. Alteration frequencies are shown to the left of the oncoplot and represent the proportion of alterations in overall cohort; counts for each alteration are given to the right. Alteration types are indicated by color as shown in the legend to the right. Patient age is displayed in a barplot at the bottom of oncoplot, as well as via violin plot to the right. **b** Cohort level copy-number plots for DAYA, GBM_RTKII, HGAP, and pedHGG_RTK1A

Among the 43 patients for whom age was known, the median age was 32 years (range 7–78) and 24 of 43 (56%) fit the definition of an Adolescent and Young Adult (AYA) patient. Of those outside of the AYA range, 4 patients were in the pediatric age group, and 13 were older; however, of the 8/13 of the older patients were between 41 and 45 years of age, close to the AYA age range (Fig. 2a). The cohort had a male predominance (29 males and 17 females). Among the 42 patients for whom tumor location was known, the supratentorial compartment predominated (30 cases, 71%), most frequently left frontal lobe, with 9 cases occurring in the cerebellum and 3 in the spinal cord (Fig. 2a, Supplementary Table 1). For many patients, radiological studies demonstrated extensive infiltration, with frequent midline and/or multiple lobe involvement.

As previously described for tumors matching to this class [19], alterations in MAPK genes, including *NF1*, *FGFR1*, *BRAF*, *PDGFRA*, and *NTRK2*, were common and were found in 29 tumors. Genomic analyses (Fig. 2a) showed a high rate of *TP53* variants (20/24 evaluable tumors) and *NF1* alterations (8/22 evaluable tumors), with *NF1* alterations always co-occurring with *TP53* variants. Mechanisms of escape from senescence were identified in only a minority of cases, with *TERT* promoter mutations identified in 4 cases, and *ATRX* variants detected in 5 cases; *TERT* promoter and *ATRX* variants were mutually exclusive, with *ATRX* variants always co-occurring with *TP53* variants. The *TERT* promoter was also noted to be remarkably hypomethylated (data not shown). Homozygous *CDKN2A*/*B* deletion was common (17/45 tumors, 37%). High-level gene amplifications, using the 29 genes displayed in the standard copy Heidelberg copy-number output format, were also common, with 22 of 46 tumors (48%) showing at least one high-level amplification among these 29 genes, and 9 of these 22 cases showing multiple (2–4) highly amplified genes. *PDGFRA* was the single most amplified gene (11/45 tumors, 24%) and additional commonly amplified genes included *MYCN* (8 cases), *MET* (4 cases), *CDK4* (3 cases), *BRAF* (3 cases), followed by *TERT* and *EGFR* (2 cases each), and *MDM4*, *CCND2*, and *CDK6* (1 case each). Although fusions were not common, they were identified in four tumors, involving *ALK*, *NTRK2* (2 cases), and *MET*.

Although the copy-number changes seen in DAYA do not allow a specific diagnosis, they do distinguish these tumors from related gliomas with distinct copy-number profiles. Specifically, for example, when examining the cohort as a whole, although subsets of cases show various gains or losses, no copy-number changes predominated throughout the cohort, in contrast to glioblastomas, where a vast majority of tumors harbor + 7/–10 (Fig. 2b). The copy-number plots for HGAP and pedHGG_RTK1A show some similarities to the copy-number plot of DAYA, but overall contrasting profiles. The copy-number plot for pedHGG_RTK1A is depicted given the closest resemblance between that subtype of pedHGG to DAYA on the unsupervised hierarchical clustering; the remaining pedHGG copy-number plots also contrast with DAYA (Supplementary Fig. 3).

**Fig. 3:**
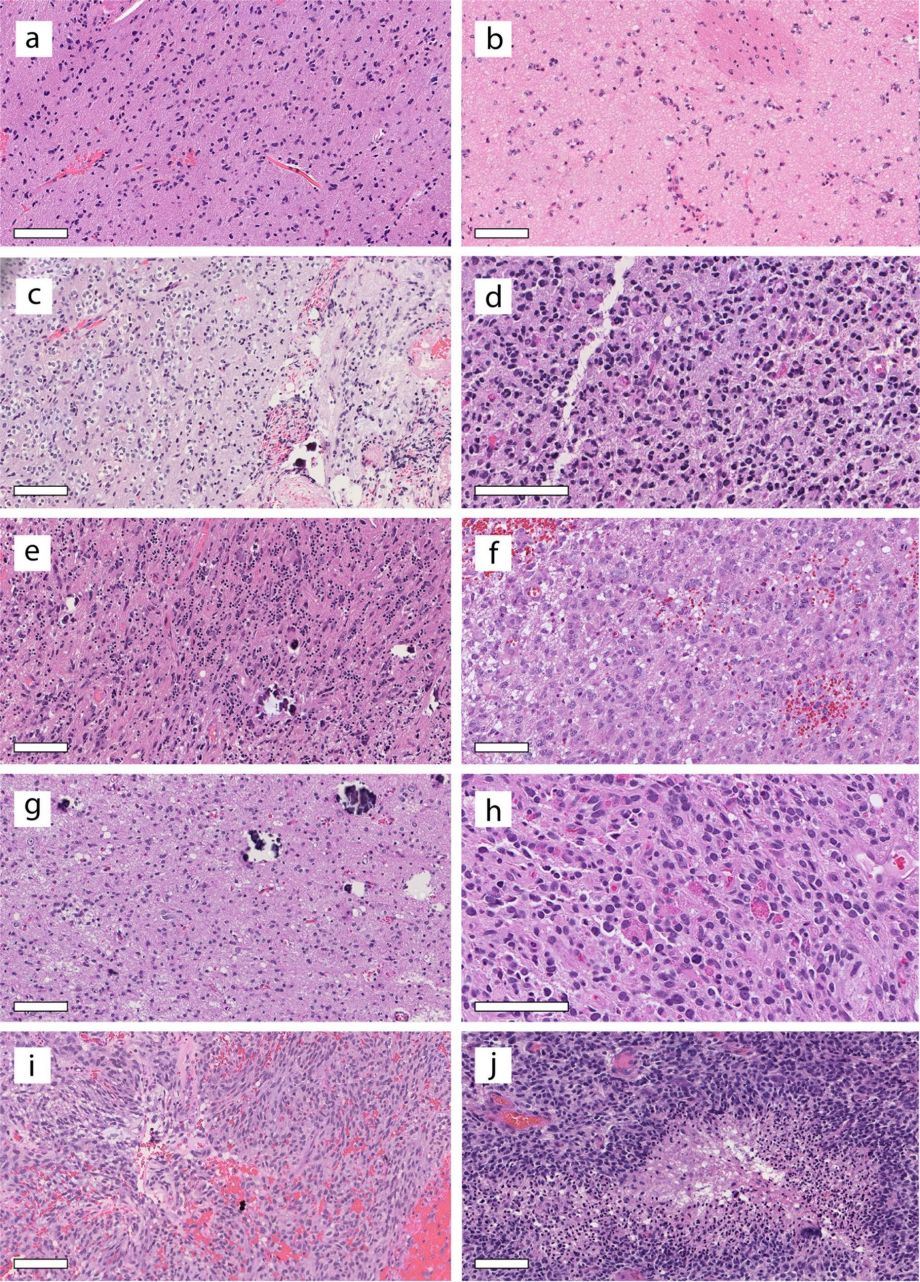
Histologic examination of DAYA demonstrates a spectrum of histologic grades and morphologic findings. **a**, **b** Some gliomas demonstrate lower cellularity and low proliferative activity, but a clear infiltrative pattern. Midline involvement is noted in a subset of cases as in (**b**), where the pencil fiber indicates the basal ganglia location. **c** A few cases demonstrated perinuclear haloes and microcalcifications, reminiscent of oligodendroglioma. **d** Epithelioid morphology with multinucleated cells was appreciated. **e** Although most cases were supratentorial, rare cases involved the cerebellum. **f** Many tumors appeared high-grade due to dense cellularity with atypical cells, but lacked necrosis and microvascular proliferation. **g**, **h** Progression of a glioma over 12 years demonstrated higher histologic grade at recurrence (**h**) compared to histology at initial presentation (**g**). **i**, **j** Additional examples of high-grade histology, including a case with the morphologic features of a GBM (**j**). Scale bars: 100 microns

Initial/preliminary (pre-methylation profiling) diagnoses and representative histopathology from the block tested by methylation were available for 32 of the cases (Supplementary Table 1). Diagnoses ranged from histologically low-grade lesions (e.g., “Ganglioglioma, “Glial neuronal tumor, favor low-grade”) to high-grade lesions (e.g., “Diffuse pediatric-type high-grade glioma”, “Glioblastoma”). Histopathologically, most cases were classified as diffuse gliomas and showed a range of histologic grades, ranging from a low-grade appearance to cases indistinguishable from glioblastomas (e.g., palisading necrosis) (Fig. 3a–j). A majority of tumors had astrocytic features, but oligodendroglioma-like cytology was present in four cases. Microvascular proliferation and/or necrosis was present in seven cases, but more common were high-grade appearing astrocytic features without necrosis or microvascular proliferation (11 cases). Dense cellularity, brisk mitotic activity, and nuclear atypia were prominent in these cases, and multinucleated cells were commonly seen but not universally present. In cases for which proliferation index was known, Ki-67 labeling ranged from very high (90%) to very low (< 1%). Immunohistochemical staining data were available for a subset of cases. Olig2 was positive in 13/13; 12/12 were positive for GFAP, although for 5 of these, the positivity was patchy (Supplementary Fig. 4). SOX10 was positive in 3/3 tumors. In addition, 13/13 had retained H3K27me3 expression; 18/19 showed retained ATRX expression. For the five cases with *ATRX* variants, immunohistochemistry was available for two cases. One case showed predominantly lost ATRX, whereas the second case showed intact ATRX expression. Synaptophysin, neurofilament, and NeuN were each performed on several cases and highlighted the infiltrative nature of the tumors, with 4/8 cases showing some positivity for synaptophysin in the tumor cells. CD34 was negative in tumor cells for 5/5 cases.

Of the two patients with matched primary-recurrent tumor pairs, one patient showed evidence of acquisition of copy-number aberrations at tumor recurrence, including development of *MET* gene amplification in the recurrent tumor. This recurrent tumor also demonstrated histopathologic tumor progression from a low-grade to a high-grade glial neoplasm (Fig. 3g, h). For the second patient with a matched primary-recurrent tumor pair, the initial histology was low grade and sequencing found no amplifications; the histology was not available for the recurrence, but again the sequencing detected no amplifications. A third recurrent tumor but without a matched primary tumor available demonstrated high-grade histology but no gene amplifications; reportedly, the initial tumor demonstrated low-grade histology. The molecular changes underlying the progression to higher grade histologic features are unknown. Despite the overall male predominance in the full cohort, the histologically low-grade tumors were found in six females and four males.

**Fig. 4:**
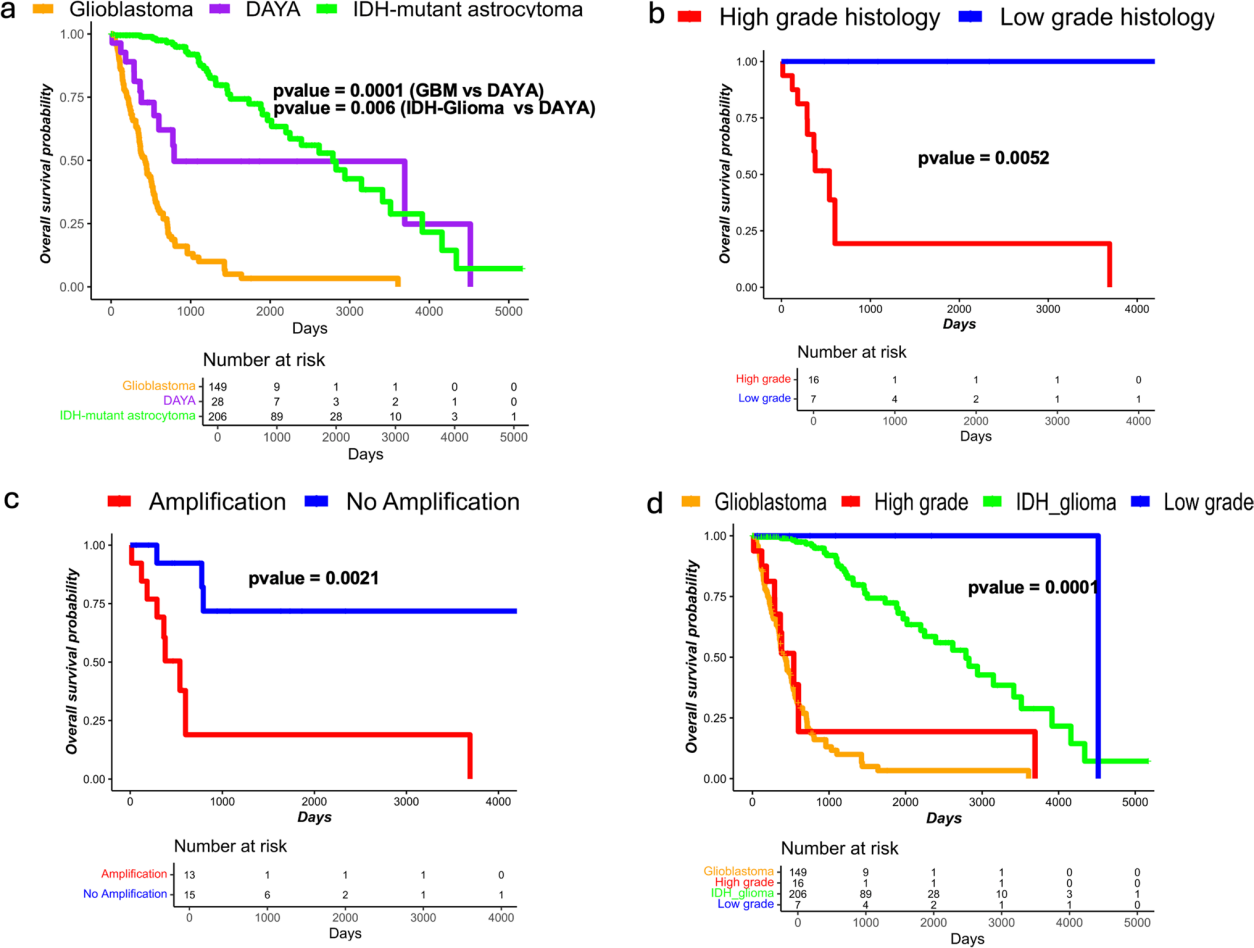
The survival plots (Kaplan–Meier curves). **a** Survival curve of the overall DAYA cohort depicted in the context of TCGA GBM and IDH-mutant astrocytoma (histologic grades 2, 3) cohorts. DAYA show intermediate outcomes. **b** When divided into histologically high-grade and histologically low-grade gliomas, survival is significantly different between the two groups (*p* = 0.0014). **c** When divided into gliomas with and without gene amplifications, survival is significantly different between the two groups (*p* = 0.0014). **d** Overlaying TCGA GBM and IDH-mutant astrocytoma curves with DAYA divided by histologic grade demonstrates that patients with high-grade histologic DAYA have outcomes similar to GBM

Outcome data were available for 30 patients. Overall survival times were intermediate between those for patients with GBMs and IDH-mutant astrocytomas, with patients having significantly improved outcomes compared to GBM patients (1.52 years versus 0.90 years), but significantly worse outcome compared to IDH-mutant astrocytoma patients (1.52 years versus 2.35 years, both *p* < 0.01, Fig. 4a). Given the range of histologic grades observed, the question of tumor prognosis stratifying with grade arose. The tumors were divided into groups with high-grade and low-grade histology, with high-grade histology defined by dense cellularity and mitotic activity sufficient for a WHO grade 3 diagnosis (i.e., for IDH-mutant astrocytoma) on the available digital slide, or microvascular proliferation, or necrosis (performed by MPN, blinded to survival). Notably, of 24 cases with high-grade histology, 20 cases showed at least one gene amplification and 10 had homozygous deletion of *CDKN2A/2B* (Supplementary Table 1). In contrast, none of the ten cases with low-grade histology demonstrated a gene amplification nor *CDKN2A/2B* homozygous deletion. These findings suggest a correlation of molecular features with histologic features. Indeed, Kaplan–Meier survival curves demonstrate significantly better survival for patients with histologically low-grade tumors compared to those with histologically high-grade tumors, as well as better outcomes for patients whose tumors had no gene amplifications compared to those with at least one gene amplification (Fig. 4b, c). Comparing the histologically stratified outcomes to TCGA cohorts of glioblastoma patients and IDH-mutant glioma patients demonstrates that the prognosis of patients with histologically high-grade tumors in the cohort is consistent with CNS WHO grade 4 (Fig. 4d). Our limited data suggest a possible grade 2 biologic behavior for the low-grade subset, but a definitive recommendation requires additional follow-up for patient outcomes.

## Discussion

Recent advances in CNS tumor classification based on DNA methylation profiling provide the opportunity to classify and diagnose tumors within existing tumor classes [4, 13, 17] as well as to explore new tumor entities that are not in the current WHO classification [12, 14, 20]. DNA methylation-based classification has prompted the integration of newly identified tumor types and subtypes into the recent edition of the WHO classification of CNS tumors. This study has been performed to provide information regarding tumor biology, as well as prognostic information to clinical teams caring for patients whose tumors fall in the Heidelberg classifier HGG_B cluster, here called DAYA. This DAYA methylation cluster has been described in the context of glioblastoma [16], and the methylation cluster was recently characterized with the proposal to recognize them as a subtype of glioblastoma with the name, “Diffuse high-grade astrocytoma, MAPK pathway-altered” [19]. The present work examines a group of additional cases that match to this methylation cluster to further characterize this epigenetically distinct type of IDH-wildtype diffuse glioma. The main diagnostic considerations that arise when assessing one of these gliomas tend to be IDH-wildtype and H3-wildtype pediatric high-grade glioma, high-grade astrocytoma with piloid features, and glioblastoma. A couple of cases with NTRK fusions are noted, which may bring other novel methylation clusters under consideration, such as GNT_KinF_A, but these cases are rare. In addition, the couple cases in the cohort with NTRK fusions also cluster tightly with DAYA cases on UMAP analysis and unsupervised hierarchical clustering.

HGAPs often show GBM-like histology and occur in young adults, with alterations in the MAPK pathway, *CDKN2A/2B* homozygous deletion, and occasionally *ATRX* variants. In these respects, HGAPs are similar to DAYA. However, HGAPs and DAYA differ in predominant location, rate of *TP53* variants, and epigenetics; the patient age distributions may differ with HGAP patients being older, but the cohorts are small, so it is not possible to confidently compare the groups.

A subset of tumors falling in the DAYA methylation cluster may be diagnosed as glioblastomas based on histology, IDH-wildtype status, and/or *TERT* promoter mutations; however, the DAYA group of patients and their tumors are distinct from glioblastomas in several respects. Although they occur in a wide age range of patients, a predominance of the patients fall in the AYA age range, rather than in the older adult range typical for glioblastomas. In addition, the tumors do not have the constellation of mutations and copy-number changes found in glioblastomas; for example, *PTEN* mutations and gain of chromosome 7 with loss of chromosome 10 are not seen, and *TERT* promoter mutations are not frequent. Given that four cases showed *TERT* promoter mutations, we note the relevance of a recent report that cautions against the presence of isolated *TERT* promoter mutation as sufficient criteria for a glioblastoma diagnosis in the setting of an IDH-wildtype diffuse glioma [15].

Finally, pediatric high-grade glioma, IDH-wildtype and H3-wildtype, tends to be a leading preliminary diagnosis prior to methylation testing given the infiltrating and commonly high-grade histology, the lack of the classic GBM genetic profile, and the younger age group. Pediatric high-grade gliomas (HGG) that are both H3- and IDH-wildtype also have high rates of *CDKN2A/2B* homozygous deletion, *MYCN* amplification, and *PDGFRA* amplification. Given that most studies of pediatric HGG focus on the pediatric age group, it is unclear how commonly patients with these tumors may be in the young adult age group. However, the spectrum of histologic grades correlating with outcomes and the distinct epigenetic clustering by unsupervised methods support that DAYA represent a separate tumor type from pediatric HGG.

Our study is limited by incomplete sequencing data and a lack of RNA expression data that could lend greater understanding to the epigenetic investigations; further sequencing and analysis will be the focus of future studies. In addition, the survival data are limited, and the possibility of grading tumors within this group requires additional study and validation. However, this study provides useful information for clinicians caring for patients with gliomas falling into the DAYA (HGG_B) DNA methylation cluster and classifying as such on the Heidelberg classifier. Identification and characterization of this methylation cluster highlight the valuable role of DNA methylation studies in tumor assessment, because although the histologic and genomic features of the tumors in the DAYA methylation cluster often do not allow a specific diagnosis based on the current WHO classification of CNS tumors, the DNA methylation profile allows the placement of these tumors in the context other gliomas, including pediatric HGG, GBM, and HGAP. Our investigations have revealed that, in addition to the shared molecular features, the tumors share a preferential age range, typical tumor location, and typical imaging features. Integration of these data suggest that gliomas in this methylation cluster may be considered “diffuse astrocytoma, adolescent and young adult-type, with MAPK alterations” (DAYA), distinct from other IDH-wildtype gliomas, with grading dependent on assessment of histologic and molecular features.

## Supplementary Information

Below is the link to the electronic supplementary material.

Supplementary file1 (TIFF 19558 KB) Supplementary Fig. 1. A heatmap derived from SNP probes verified uniqueness of the samples, with the only two sets of duplicates representing two patients for whom both primary and recurrent tumors were studied

Supplementary file2 (TIF 24675 KB) Supplementary Fig. 2. Differential promoter analysis of DAYA with GBMs, pediatric HGG, and HGAP. Volcano plots showed the log fold change on x axis and -log10P value on y axis. Significant change shown in red (--log10P>20, log2FC>4)

Supplementary file3 (TIF 24677 KB) Supplementary Fig. 3. Cohort level copy number plots for additional pediatric high-grade glioma subtypes

Supplementary file4 (TIF 12755 KB) Supplementary Fig. 4. Representative immunohistochemical staining. GFAP was positive in 12/12 cases, but in almost half of cases it was patchy. Olig2 was positive in 13/13 cases. Synaptophysin highlighted the infiltrative nature of the tumors, with 4/8 cases. 18/19 cases showed retained ATRX expression. Scale bar 100 microns in lower left corner of hematoxylin and eosin-stained section image applies to all images

Supplementary file5 (XLSX 26 KB)

## Data Availability

Data are provided within the manuscript or supplementary information files.
