# Supplementary figures and images for: Diffuse astrocytoma, AYA-type, frequently MAPK-altered: report of 45 patients
Source: Acta Neuropathol. 2025 Apr 9;149(1):32. doi: 10.1007/s00401-025-02873-8 (PMC11982118; doi:10.1007/s00401-025-02873-8)

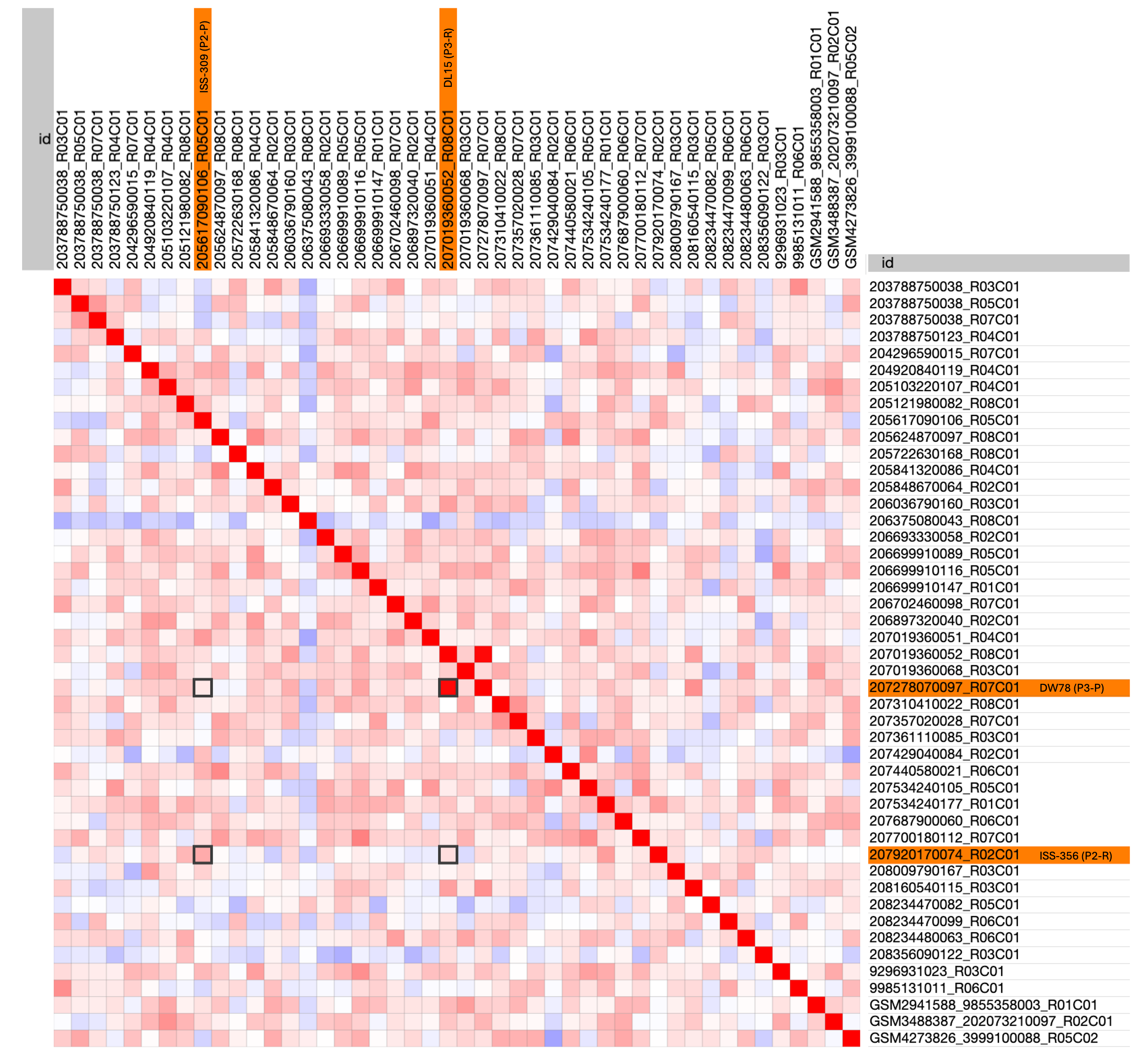

Supplement: Supplementary file 1 — Supplementary file1 (TIFF 19558 KB) Supplementary Fig. 1. A heatmap derived from SNP probes verified uniqueness of the samples, with the only two sets of duplicates representing two patients for whom both primary and recurrent tumors were studied [file 401_2025_2873_MOESM1_ESM.tiff]

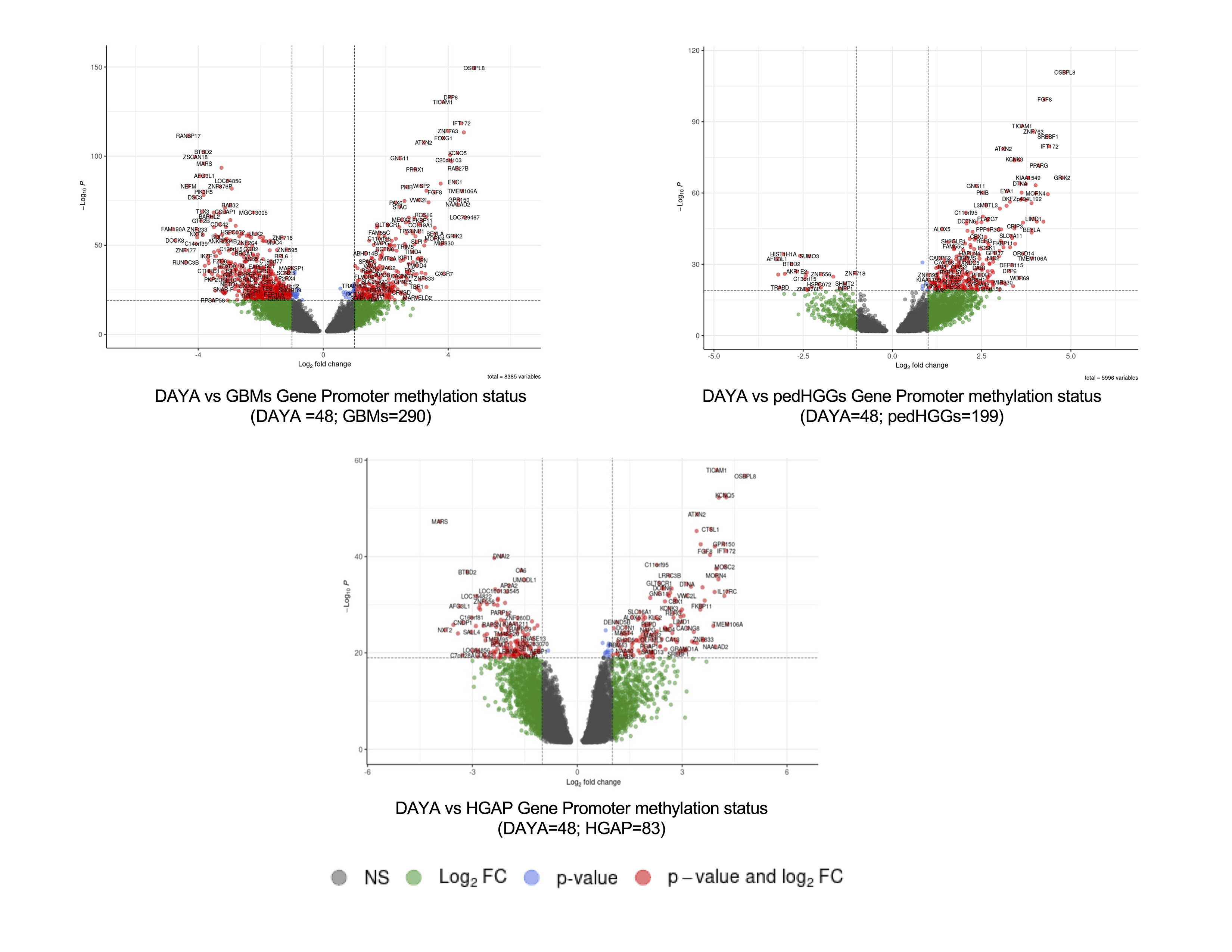

Supplement: Supplementary file 2 — Supplementary file2 (TIF 24675 KB) Supplementary Fig. 2. Differential promoter analysis of DAYA with GBMs, pediatric HGG, and HGAP. Volcano plots showed the log fold change on x axis and -log10P value on y axis. Significant change shown in red (--log10P>20, log2FC>4) [file 401_2025_2873_MOESM2_ESM.tif]

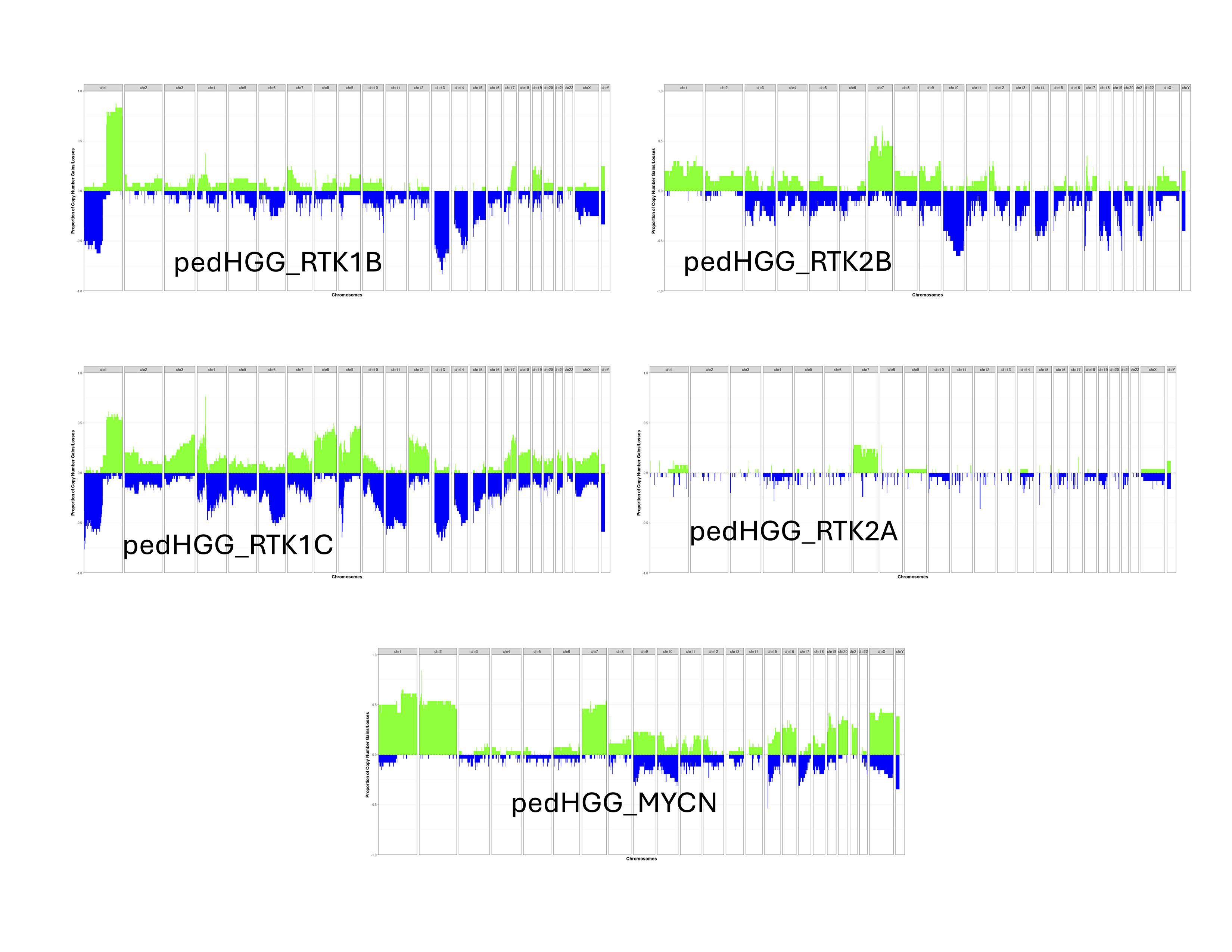

Supplement: Supplementary file 3 — Supplementary file3 (TIF 24677 KB) Supplementary Fig. 3. Cohort level copy number plots for additional pediatric high-grade glioma subtypes [file 401_2025_2873_MOESM3_ESM.tif]

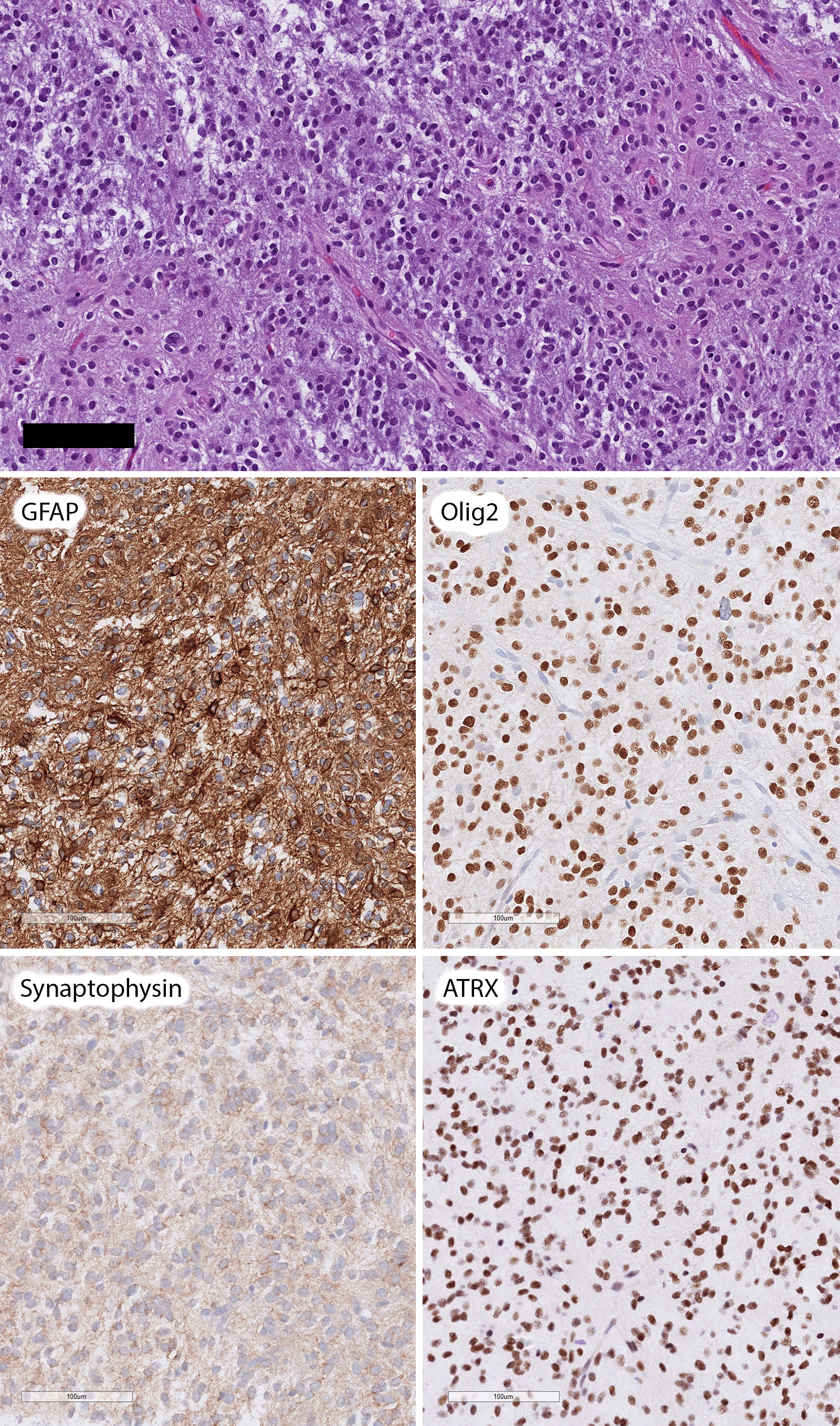

Supplement: Supplementary file 4 — Supplementary file4 (TIF 12755 KB) Supplementary Fig. 4. Representative immunohistochemical staining. GFAP was positive in 12/12 cases, but in almost half of cases it was patchy. Olig2 was positive in 13/13 cases. Synaptophysin highlighted the infiltrative nature of the tumors, with 4/8 cases. 18/19 cases showed retained ATRX expression. Scale bar 100 microns in lower left corner of hematoxylin and eosin-stained section image applies to all images [file 401_2025_2873_MOESM4_ESM.tif]
